# Reliability and Validity of Power Spectrum Slope (PSS): A Metric for Measuring Resting-State Functional Magnetic Resonance Imaging Activity of Single Voxels

**DOI:** 10.3389/fnins.2022.871609

**Published:** 2022-05-06

**Authors:** Zhenxiang Zang, Yang Qiao, Shaozhen Yan, Jie Lu

**Affiliations:** ^1^Department of Radiology and Nuclear Medicine, Xuanwu Hospital, Capital Medical University, Beijing, China; ^2^Beijing Key Laboratory of Magnetic Resonance Imaging and Brain Informatics, Beijing, China; ^3^Center for Cognition and Brain Disorders, The Affiliated Hospital of Hangzhou Normal University, Hangzhou, China

**Keywords:** power spectrum slope, resting-state fMRI, eyes closed and open, ALFF, test–retest reliability

## Abstract

Methods that capture the features of single voxels of resting-state fMRI (RS-fMRI) could precisely localize the abnormal spontaneous activity and hence guide precise brain stimulation. As one of these metrics, the amplitude of low-frequency fluctuation (ALFF) has been used in numerous studies, however, it is frequency-dependent and the division of frequency bands is still controversial. Based on the well-accepted power law of time series, this study proposed an approach, namely, power spectrum slope (PSS), to characterize the RS-fMRI time series of single voxels. Two metrics, i.e., linear coefficient b and power-law slope b’ were used and compared with ALFF. The reliability and validity of the PSS approach were evaluated on public RS-fMRI datasets (*n* = 145 in total) of eyes closed (EC) and eyes open (EO) conditions after image preprocessing, with 21 subjects scanned two times for test–retest reliability analyses. Specifically, we used the paired *t*-test between EC and EO conditions to assess the validity and intra-class correlation (ICC) to assess the reliability. The results included the following: (1) PSS detected similar spatial patterns of validity (i.e., EC–EO differences) and less test–retest reliability with those of ALFF; (2) PSS linear coefficient b showed better validity and reliability than power-law slope b’; (3) While the PPS showed less validity in most regions, PSS linear coefficient b showed exclusive EC–EO difference in the medial temporal lobe which did not show in ALFF. The power spectrum plot in the parahippocampus showed a “cross-over” of power magnitudes between EC and EO conditions in the higher frequency bands (>0.1 Hz). These results demonstrated that PSS (linear coefficient b) is complementary to ALFF for detecting the local spontaneous activity.

## Introduction

One of the potential clinical translations of resting-state functional magnetic resonance imaging (RS-fMRI) is the precise localization of abnormal brain activity which would help not only the qualitative diagnosis but also guide precise stimulation therapy, e.g., deep brain stimulation and transcranial magnetic stimulation. Among the countless analytic methods for RS-fMRI, only a limited number of methods have been developed for precise localization of the abnormal brain activity and further have been included in coordinate-based or voxel-based meta-analyses. For example, a single voxel-level metric, the amplitude of low-frequency fluctuation (ALFF) (Zang et al., 2007), and its derivative fractional ALFF (fALFF) (Zou et al., 2008) can reliably reveal abnormal brain activities in a few meta-analytic studies, e.g., decreased ALFF in the putamen of Parkinson’s disease (PD) (Wang et al., 2018; Jia et al., 2021) and the posterior cingulate cortex of patients with mild cognitive impairment (MCI) (Pan et al., 2017) which were very consistent with gold-standard with positron emission tomography (PET) showing decreased dopamine in the putamen in PD (Niccolini et al., 2014) and increased deposition of β amyloid in alzheimer’s disease (AD) (He et al., 2015), respectively.

While ALFF was frequently used to measure the brain’s low-frequency fluctuations (e.g., 0.01–0.1 Hz), signal oscillations of the brain are integrated with multiple frequency bands (Buzsáki and Draguhn, 2004; Buzsáki et al., 2013), suggesting that investigation of how brain signals fluctuate in different frequency bands is also critical to reveal the neural basis of the brain. Similar to frequency-dependent analyses of the electrophysiological signal, i.e., frequency bins such as the α, β, and θ bands, the resting-state BOLD signal also fluctuates differently in different frequency bands. Since Zuo et al. (2010) decomposed the RS-fMRI signals into multiple frequency intervals using the N3L theory (e.g., 0.01–0.027 Hz or slow-5, 0.027–0.073 Hz or slow-4, 0.073–0.198 Hz or slow-3, 0.198–0.25 Hz or slow-2), an increasing number of studies have applied such approaches to investigate frequency-specific oscillations in different conditions (Yuan et al., 2014) and diseases (Han et al., 2011; Mascali et al., 2015; Zhao et al., 2015).

However, while most RS-fMRI studies on multiple frequency bands have applied the bins proposed by Zuo et al. (2010), some studies divided the entire frequency band into 3 (Malinen et al., 2010) or 6 (Otti et al., 2013) equal parts. Different frequency intervals may cause difficulties for group-level statistical analysis and result interpretation. An integrated method that directly captures the amplitude of multiple frequency bands is needed.

It has been widely reported that the electroencephalography (EEG) signals are powerlaw alike (Henrie and Shapley, 2005; Fransson et al., 2013; Lei et al., 2015; Pathania et al., 2021). A few RS-fMRI have also used power law as a biomarker (He, 2011; Fransson et al., 2013; Lei et al., 2015). However, no study has investigated reliability and validity.

Previous studies have shed light on the power-shift of task conditions compared to the resting-state condition (Baria et al., 2011; He, 2011). Both the studies have concluded that the brain oscillations along the different frequency bins are distributed differently between task and resting state. Indicatively, therefore, the slope of the power decay of the brain signal may be a representative measure of brain oscillation distribution. The current study aimed to validate the power spectrum slope (PSS) with two metrics, i.e., linear coefficient b and the power-law slope b’, of the power spectrum in public RS-fMRI datasets with eyes open (EO) and eyes closed (EC) states because the difference between EC and EO has shown to be very reproducible. Considering that ALFF has been widely used, we compared the PSS indices with ALFF, including its test–retest reliability and validity.

## Materials and Methods

### Subjects

The public data were nine RS-fMRI datasets under EC and EO conditions.^1^ The RS-fMRI datasets were approved by the local ethic committees. Among the 187 right-handed subjects in total, we included 146 subjects from 7 datasets with TR = 2,000 ms (age 22.9 ± 2.2 SD, 70 men). We did not use the datasets with TR = 400 ms because the PSS would be very different from that of TR = 2,000 ms. The order of EC and EO scanning sessions was counterbalanced. Specifically, subjects from datasets 1–3 (*n* = 21) underwent three scans for test–retest reliability investigation (Yuan et al., 2018; Zhao et al., 2018). Data from the first two visits were acquired on a GE scanner, and data from the third visit were acquired on a Siemens scanner. We used only the first two visits to measure the intra-scanner reliability in this study because of the limitation of order effect for the “inter-scanner” of that dataset (refer to Section “Test–Retest Reliability”).

### Resting-State Functional Magnetic Resonance Imaging Acquisition Parameters

Detailed information on the RS-fMRI acquisition is provided on https://www.nitrc.org/projects/eceo_rsfmri_9/. Here, we listed a summary of the acquisition parameters in Supplementary Table 1. All RS-fMRI data that we used in this study were acquired on GE 750 3T scanner.

### Resting-State Functional Magnetic Resonance Imaging Data Preprocessing

Statistical parametric mapping (SPM12) was used to preprocess the RS-fMRI images. After converting from DICOM to NIFTI format, RS-fMRI images were preprocessed for head motion correction, slice timing, coregistering to the high-resolution 3D T1 images, segmentation of tissue possibility templates, and spatial normalization *via* T1 images (resampled to 3 mm^3^ × 3 mm^3^ × 3 mm^3^). The normalized functional images were then spatially smoothed with an 8-mm FWHM Gaussian kernel. After spatial smoothing, we further regressed out the time series of white matter (WM 99% probability SPM map), cerebrospinal fluid (CSF 90% probability SPM map), global mean time course, six head motion parameters from the realignment step, and the frame-wise displacement (FD) (Power et al., 2012). One subject was excluded due to inaccurate spatial normalization, leaving 145 subjects for analysis.

### Fitting Power Spectrum Slope

After image preprocessing, the power spectrum of each voxel’s time series was produced *via* fast Fourier transformation (FFT). The very low frequency (<0.01 Hz) was filtered out. The upper-most frequency for 2,000-ms TR datasets is 0.25 Hz. Before fitting, we divided the power of each voxel’s signal by the average amplitude across the chosen frequency range (from 0.01 to 0.25 Hz) to normalize the scale of power. We computed both the linear coefficient and the slope of power-law fit. For linear coefficient, we used


(1) y=bx+a

where y is the normalized amplitude of the signal power after FFT, x is the corresponding frequency bin (e.g., from 0.01 to 0.25 Hz) and b is the linear coefficient.

For power-law fit, we used


(2) $$y=a^{\prime }x^{b^{\prime }}$$

This equation can be transformed into:


(3) $$\text{ln}\left(y\right)=\text{ln}\left(a^{\prime }\right)+b^{\prime }\text{ln}\left(x\right)$$

where the slope b’ can be solved *via* the least-square equation.

**FIGURE 1:**
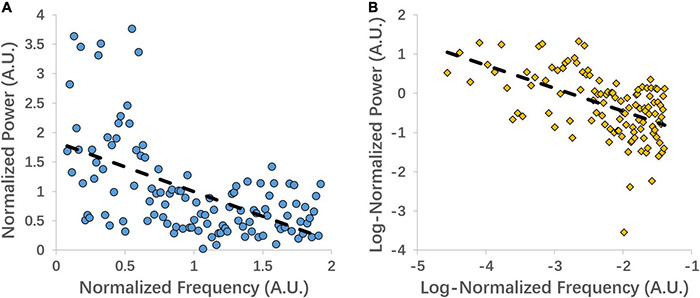
An example of linear coefficient b **(A)** and power-law slope b’ **(B)** regression fitting from the left precentral gyrus of one subject.

Both the linear coefficient b and the power-law slope b’ (i.e., non-linear slope) can represent the power decay from low to high frequency, with the more negative value representing steeper decay. For illustration purposes, we extracted the signal from the left precentral gyrus of one subject and show the linear and power-law (log) regression fittings (Figure 1).

For comparisons between the fitted PSS and ALFF, we, in addition, calculated ALFF of 0.01–0.25 Hz and the conventional band of 0.01–0.1 Hz.

### Standardization Across Datasets: Group-Level Z- and Individual-Level Z-Transformation

Considering the multi-datasets, we normalized the PSS metrics and also the ALFF using the group-level Z-transformation for each subject (Kassraian-Fard et al., 2016). In detail, we pooled the EC and EO conditions together for each voxel within each dataset and then applied the following transformation for each voxel:


(4) $$Z_{group}=\frac{rawvalue-dataset^{\prime }mean}{dataset^{\prime }sstandarddeviation}$$

In this formula, the dataset’s mean and standard deviation represent the mean and standard deviation, respectively, of a given voxel across all subjects (EC and EO conditions) in each dataset.

In addition, we tested the effect of individual-level Z-transformation using a similar equation:


(5) $$Z_{individual}=\frac{rawvalue-\left(individualglobalmean\right)}{individualstandarddeviation}$$

where the individual’s mean and standard deviation represent the mean and the standard deviation, respectively, within the whole-brain mask.

### Validity: Paired *T*-Test of Eyes Closed vs. Eyes Open

We performed paired *t*-tests on the four metrics (PSS: linear coefficient b and power-law slop*e* b’, ALFF 0.01–0.01 Hz, and ALFF 0.01–0.25 Hz). Voxels were considered significant after surviving a whole-brain two-tailed Gaussian random field (GRF) correction with cluster’s *p* < 0.05 and voxel’s *p* < 0.001. The GRF correction was performed using the DPABI software (Yan et al., 2016).

### Test–Retest Reliability

Datasets 1–3 had 3 visits of the same group of subjects. Visit 1 (V-1) and visit 2 (V-2) are from the same scanner with about 2 weeks apart. Visit 3 (V-3) is from another scanner about 8 months after V-3. The reliability analysis between V-2 and V-3 has been taken as inter-scanner reliability (Yuan et al., 2018; Zhao et al., 2018). But a limitation is that there is an order effect, i.e., V2 is always before V3. Therefore, the reliability between V2 and V3 is a mixed effect of test–retest reliability and inter-scanner reliability. Therefore, the current study analyzed test–retest reliability between V-1 and V-2 only. The test–retest reliability was estimated by using intra-class correlation (ICC) for both the EC and EO conditions as in the following equation (Shrout and Fleiss, 1979):


(6) $$ICC=\frac{MSb-MSw}{MSB+\left(K-1\right)MSw}$$

where *MSb* is between-subject effect, *MSw* is within-subject effect, and *K* represents the number of sessions (*K* = 2 here).

### Graphical User Interface of Power Spectrum Slope

**FIGURE 2:**
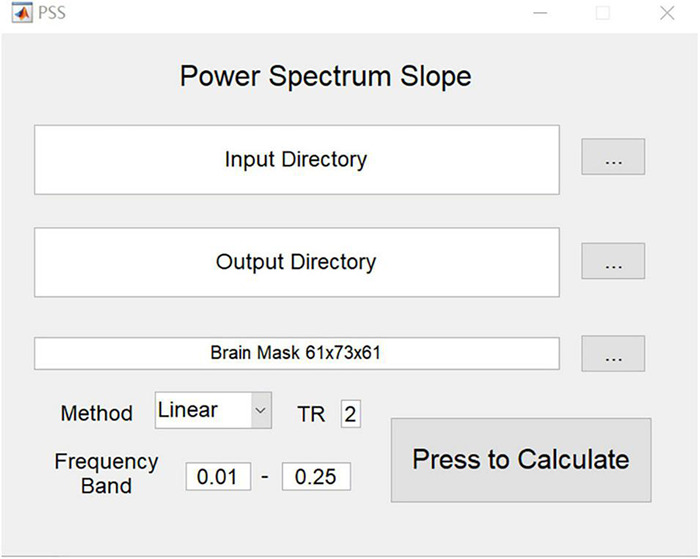
GUI of PSS. Input directory requires the arranged folder of each subject (e.g., subj001, subj002…). In each subject’s folder, only one preprocessed 4-D file is provided. Please note that filtering is forbidden. PSS toolkit will generate a pair of output images for each subject: the original coefficient image and the individual Z-transformed image.

To assist in future application studies, we implemented a graphical user interface (GUI) toolkit for PSS on RESTplus (Jia et al., 2019) and MATLAB (Figure 2).

### Goodness of Fit

We tested the goodness-of-fit (GoF) of both the linear coefficient b and power-law slope b’ using the residuals of regression without Z-transformations:


(7) $$GoF=1-\frac{\sum {\left(\hat{y}-\bar{y}\right)}^{2}}{\sum {\left(y-\bar{y}\right)}^{2}}$$

where $\hat{y}$ is the fitted data, $\bar{y}$ is the mean of the data, and y is the real data.

## Results

### Computational Validation of the Power Spectrum Slope Graphical User Interface

Power spectrum slope graphical user interface (PSS GUI) was implemented on MATLAB and RESTplus. We compared the output images of the PSS GUI using both the linear coefficient b and the power-law slope b’ approaches with the prior-generated images (codes without GUI) of EC and EO conditions. The GUI’s output images were identical to the prior-generated images for both approaches and conditions.

### Power Spectrum Slope Distribution in the Brain

**FIGURE 3:**
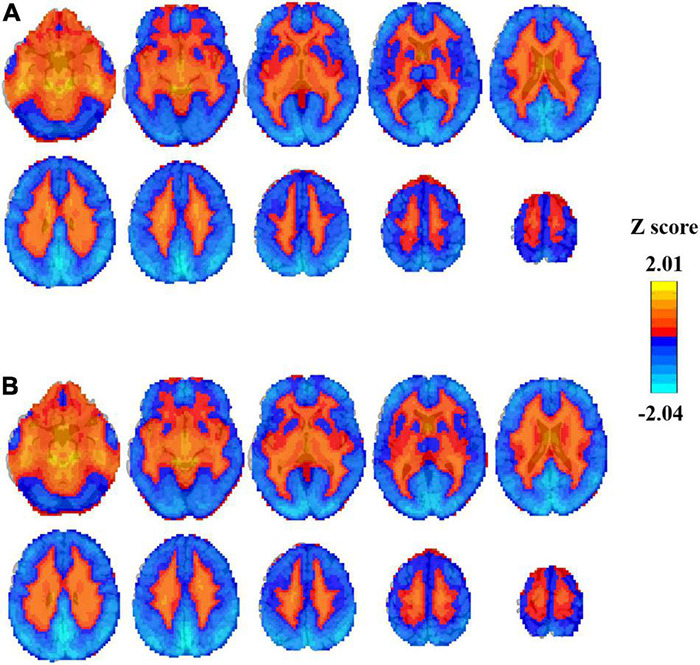
Individual Z-transformed PSS (linear coefficient b) distribution in EC **(A)** and EO **(B)** conditions averaged across all subjects (*n* = 145).

Here, we show the average Z-score of the PSS in the brain (Figure 3). For both the EC and EO conditions, the majority of the cerebral cortex, especially in the visual area, showed negative Z values of PSS (i.e., steeper slope than the global mean PPS). Positive Z-PSS values were found mainly in the white matter and ventricles.

### Power Spectrum Slope and Amplitude of Low-Frequency Fluctuation Difference Between Eyes Closed and Eyes Open Using Group Z-Transformation

**FIGURE 4:**
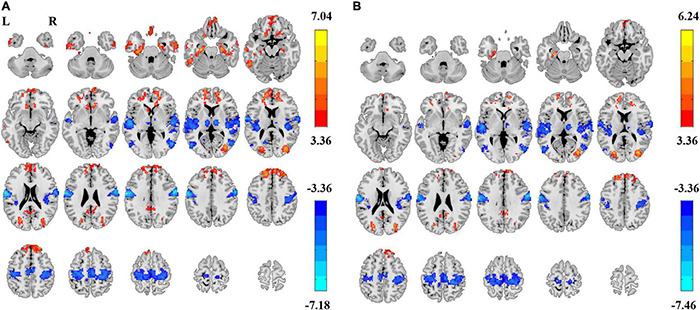
Differences of power spectrum slope (PSS) between EC and EO using group-level Z-transformed coefficient b and power-law slope b’. Significant difference between EC and EO conditions using coefficient b (GRF correction, *P*_corrected_ < 0.05, |T| > 3.36, cluster size > 40 voxels) **(A)** and using power-law slope b’ (GRF correction, *P*_corrected_ < 0.05, |T| > 3.36, cluster size > 50 voxels) **(B)**. L, left; R, right.

In summary, both the linear coefficient b and the power-law slope b’ showed steeper PSS (i.e., faster decay) in EC than EO conditions in the sensorimotor area, thalamus, and visual-parietal areas. Less steep PSS in EC than EO conditions was found in the visual area and the default mode network (Figure 4). Comparably, the linear coefficient b approach provided more voxels that survived GRF correction (|T| > 3.36, cluster size > 40 voxels, 5,854 voxels in total) than the power-law slope b’ (|T| > 3.36, cluster size > 50 voxels, 3,839 voxels in total). Several clusters in the limbic system (e.g., hippocampus, amygdala, and ventral striatum) survived GRF correction using the linear coefficient b but not the power-law slope b’. The detailed information on paired *t*-test using the linear coefficient b is provided in Table 1.

**TABLE 1:** Location of peak voxels that showed significant difference between EC and EO condition using linear coefficient b.

Peak location	Peak MNI	Peak T	Cluster size (voxels)
R. Fusiform (BA 20)	36, −3, −45	5.46	48
L. Middle Temporal (BA 21)	−54, 0, −27	4.58	153
R. Parahippocampa (BA 35)	24, −15, −21	5.58	238
L. Parahippocampa (BA 20)	−27, −18, −24	7.04	166
R. Rectus*	−3, 24, −30*	5.54	604
L. Inferior Temporal (BA 20)	−51, −51, −18	5.20	88
L. Medial.Sup. Frontal (BA 10)	−12, 54, 15	5.69	926
R. Postcentral (BA 4)	60, −3, 36	–7.18	2,875
R. Middle Temporal (BA 37)	51, −69, 3	–5.73	138
R. Middle Occipital (BA 19)	33, −78, 12	6.17	195
L. Middle Occipital (BA 37)	−51, −72, 9	–5.72	103
L. Thalamus	−12, −15, 6	–5.21	53
R. Thalamus	9, −15, 9	–5.33	54
L. Middle Occipital (BA 19)	−24, −75, 18	5.34	107
L. Precuneus (BA 23)	−9, −54, 24	4.17	106

Information on the table was provided by xjviewer (https://www.alivelearn.net/xjview/). The name of the corresponding brain area of the peak voxels was provided based on the automated anatomical labeling atlas (AAL) and the Brodmann area (BA). *The peak voxel does not fall in any brain region in AAL, and we, therefore, reported the nearest brain area. R, right; L, left.

**FIGURE 5:**
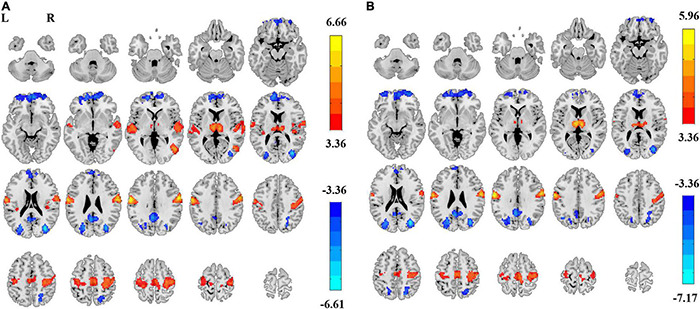
ALFF differences between EC and EO. Significant difference of ALFF between EC and EO conditions in frequency bands of 0.01–0.1 Hz [Panel **(A)**, GRF correction, *P*_corrected_ < 0.05, |T| > 3.36, cluster size > 89 voxels] and 0.01–0.25 Hz [Panel **(B)**, GRF correction, *P*_corrected_ < 0.05, |T| > 3.36, cluster size > 83 voxels]. L, left; R, right.

Paired *t*-tests on ALFF also showed significant differences in EC vs. EO conditions in both the 0.01–0.1 Hz frequency band (GRF corrected, |T| > 3.36, cluster size > 89 voxels, Figure 5A) and 0.01–0.25 Hz frequency band (GRF corrected, |T| > 3.36, cluster size > 83 voxels, Figure 5B). The spatial pattern of ALFF was similar to that of PSS. The magnitude of T values in the brain was opposite in ALFF than that in PSS.

**FIGURE 6:**
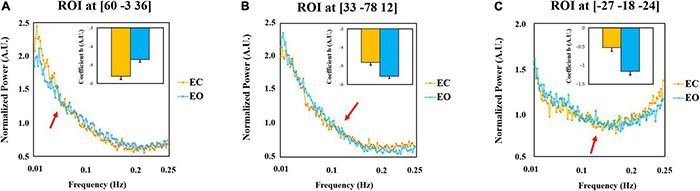
*Post-hoc* plots of the power spectrum and the linear coefficient *b* and the normalized power amplitude of EC and EO conditions. The mean power spectrum and slope of all subjects in EC and EO conditions in the sensorimotor cluster (MNI = [60 –3 36]) **(A)**, the occipital cluster (MNI = [33 –78 12]) **(B)**, and the parahippocampus (MNI = [–27 –18 –24]) **(C)**. The difference in the sensorimotor cortex [EC > EO, Panel **(A)**] and visual cortex [EO > EC, Panel **(B)**] is mainly contributed by the lower frequency band. However, the difference in the parahippocampus [EC > EO, Panel **(C)**] is mainly in the higher frequency band. The plots represent averaged values across 145 subjects. Error bars represent standard errors. AU refers to the arbitrary unit. The arrows are the indicators of the location where the power spectrum “cross-over” effect appears.

We extracted the mean power spectrum and linear coefficient b of all subjects in EC and EO conditions in three representative spherical regions of interest (ROI) (radium = 5 mm) centered at the peak voxel in the sensorimotor cluster (MNI = [60 −3 36]), the occipital cluster (MNI = [33 −78 12]), and the parahippocampus (MNI = [−27 −18 −24]). The results are shown in Figure 6. For the selected three ROIs, “cross-over” effects of the power spectrum between EC and EO can be obtained (red arrows).

### Z-Transformation in the Individual Space Provides Similar Eyes Closed–Eyes Open Differences

**FIGURE 7:**
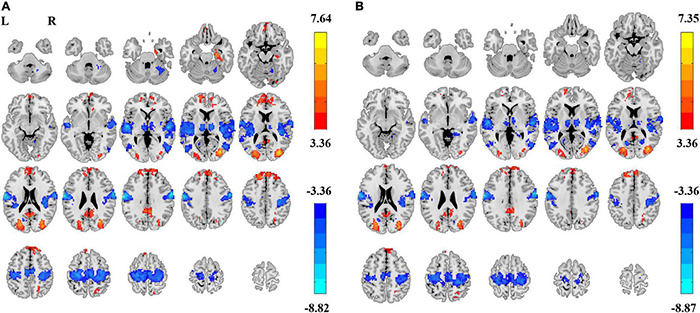
PSS differences between EC and EO conditions using individual-level Z-transformed coefficient b and power-law slope b’. Significant difference between EC and EO conditions using individual-level Z-transformed coefficient b [Panel **(A)**, GRF correction, *P*_corrected_ < 0.05, |T| > 3.36, cluster size > 75 voxels] and using power-law slope b’ [Panel **(B)**, GRF correction, *P*_corrected_ < 0.05, |T| > 3.36, cluster size > 51 voxels]. L, left; R, right.

We tested the effect of individual-space Z-transformation. Paired *t*-tests revealed highly similar EC–EO differences (Figure 7) as the group-level Z-transformation did.

**FIGURE 8:**
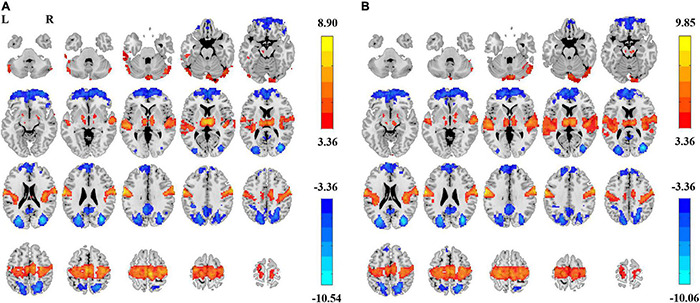
EC–EO difference of ALFF with individual-level Z-transformation. Significant difference between EC and EO conditions of individual-level Z-transformation of ALFF in low-frequency band (0.01–0.1 Hz) [Panel **(A)**, GRF correction, *P*_corrected_ < 0.05, |T| > 3.36, cluster size > 214 voxels] and in full frequency band (0.01–0.25 Hz) [Panel **(B)**, GRF correction, *P*_corrected_ < 0.05, |T| > 3.36, cluster size > 223 voxels]. L, left; R, right.

Comparably, Z-transformation in the individual space for ALFF provided much more significant voxels of EC–EO difference. For ALFF (0.01–0.1 Hz), 11,505 voxels survived GRF correction (Figure 8A). For ALFF (0.01–0.25 Hz), 10,613 voxels survived GRF correction (Figure 8B).

**FIGURE 9:**
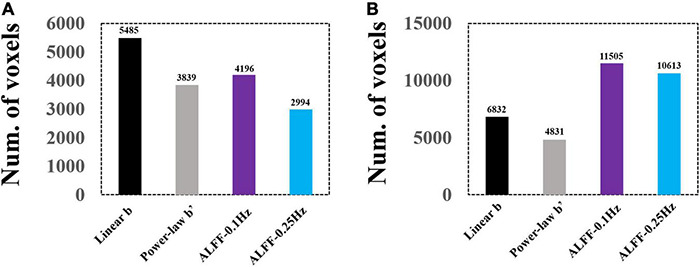
Voxel numbers of GRF-corrected differences between EC and EO conditions using group and individual Z-transformation. Panel **(A)** shows the number of voxels surviving GRF correction for group Z-transformation. Panel **(B)** shows the number of voxels surviving GRF correction for individual Z-transformation.

A direct comparison of the number of voxels that survived GRF correction is shown in Figure 9.

### Spatial Overlap of Eyes Closed–Eyes Open Differences Between Power Spectrum Slope and Amplitude of Low-Frequency Fluctuation

**FIGURE 10:**
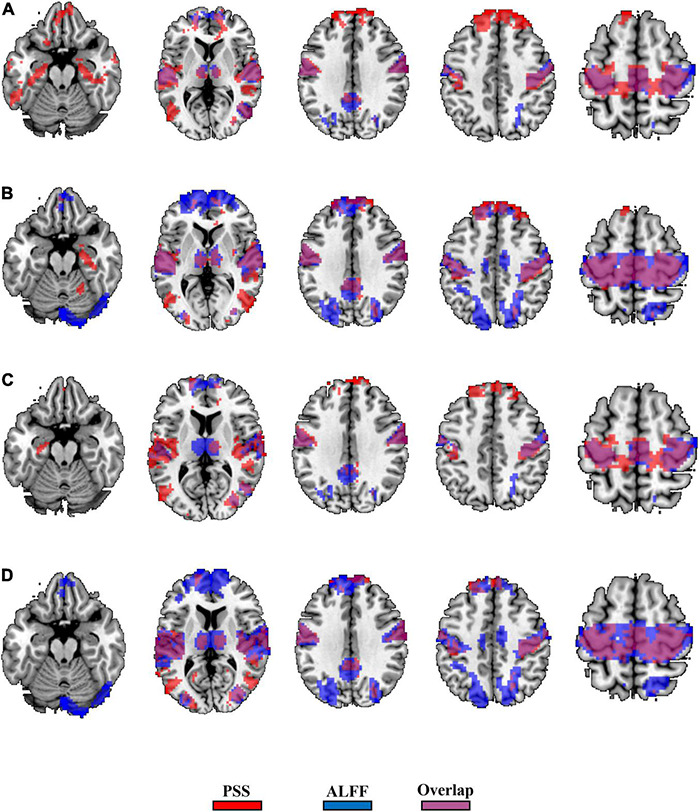
Binary spatial overlap between the EC–EO differences using the PSS and ALFF (0.01–0.1 Hz). Panel **(A)** shows the spatial overlap of the EC–EO difference using the PSS (linear coefficient b) and ALFF (0.01–0.1 Hz) with group Z-transformation. Panel **(B)** shows the spatial overlap of the EC–EO difference using the linear coefficient b and ALFF (0.01–0.1 Hz) with individual Z-transformation. Panel **(C)** shows the spatial overlap of the EC–EO difference using the PSS (power-law slope b’) and ALFF (0.01–0.1 Hz) with group Z-transformation. Panel **(D)** shows the spatial overlap of the EC–EO difference using the PSS (power-law slope b’) and ALFF (0.01–0.1 Hz) with individual Z-transformation.

**FIGURE 11:**
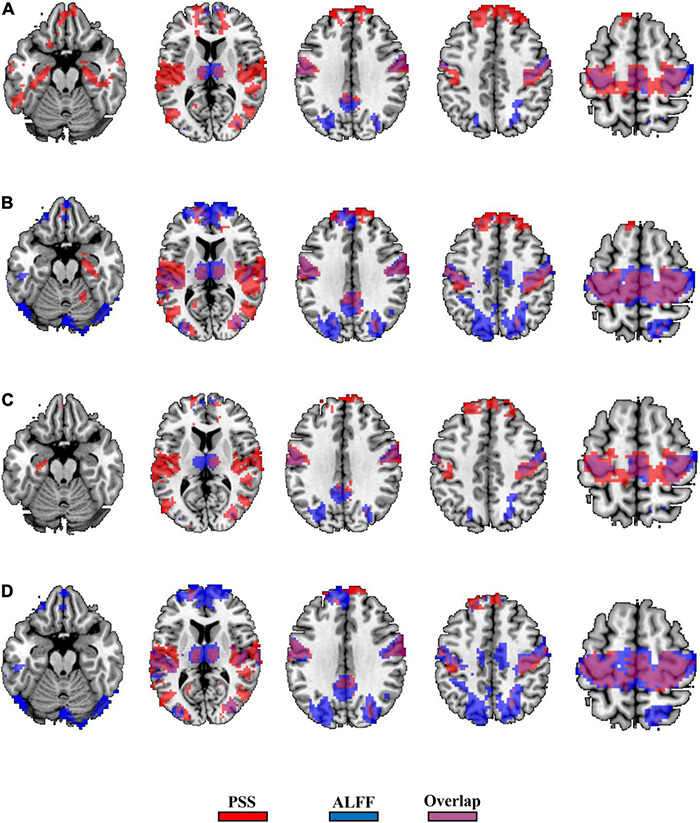
Binary spatial overlap between the EC–EO differences using PSS and ALFF (0.01–0.25 Hz). Panel **(A)** shows the spatial overlap of the EC–EO difference using the PSS (linear coefficient b) and ALFF (0.01–0.25 Hz) with group Z-transformation. Panel **(B)** shows the spatial overlap of the EC–EO difference using the linear coefficient b and ALFF (0.01–0.25 Hz) with individual Z-transformation. Panel **(C)** shows the spatial overlap of the EC–EO difference using the PSS (power-law slope b’) and ALFF (0.01–0.25 Hz) with group Z-transformation. Panel **(D)** shows the spatial overlap of the EC–EO difference using the PSS (power-law slope b’) and ALFF (0.01–0.25 Hz) with individual Z-transformation.

The GRF corrections for each paired *t*-test yield different cluster sizes, causing difficulty for direct comparison between the validity of the PSS and ALFF approaches. We thus used *P*_uncorrected_ < 0.001 (|T| > 3.36) and cluster size > 10 voxels as the same threshold and generated the binary brain maps for PSS (linear coefficient b) and ALFF (0.01–0.1 Hz as well as 0.01–0.25 Hz). As a result, 36.7% of the voxels (2,819/7,672) overlapped between the EC–EO difference using the linear coefficient b and ALFF (0.01–0.1 Hz, Figure 10A) with the group Z-transformation. Similarly, 43.9% of the voxels (5,598/12,739) overlapped between the EC–EO difference using the linear coefficient b and ALFF (0.01–0.1 Hz, Figure 10B) with the individual Z-transformation. The spatial overlap between the power-law slope b’ and ALFF (0.01–0.1 Hz) is shown in Figures 10C,D. Spatial overlap between PSS and ALFF (0.01–0.25 Hz) is shown in Figure 11. PSS (linear coefficient b) presented exclusive results in the parahippocampus which was not found by either ALFF of 0.01–0.1 Hz or 0.01–0.25 Hz. In addition, we generated the PSS–ALFF contrast mask to specifically show the difference in Supplementary Figure 1 (ALFF 0.01–0.1 Hz) and Supplementary Figure 2 (ALFF 0.01–0.25 Hz).

We calculated the voxel-wise intra-subject correlation between the PSS and the ALFF (0.01–0.1 and 0.25 Hz). Voxels in the brainstem, medial temporal gyrus, and prefrontal gyrus showed a positive correlation between PSS and ALFF during EC but not EO condition. The rest majority of voxels showed a negative correlation (Supplementary Figures 3–6).

### Test–Retest Reliability for Eyes Closed and Eyes Open Using Group Z-Transformation

**FIGURE 12:**
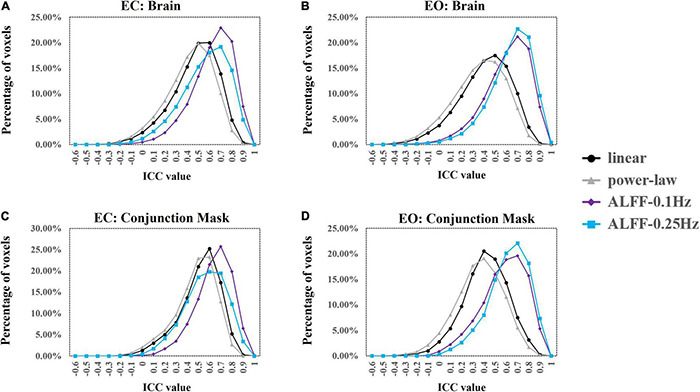
ICC histograms of coefficient b and power-law slope b’ and ALFF under EC and EO conditions using group Z-transformation. Panels **(A,B)** show the histograms of ICC values of EC **(A)** and EO **(B)** conditions from the entire brain. Panels **(C,D)** show the histograms of ICC values of EC **(C)** and EO **(D)** conditions from the conjunction mask of the voxels that survived GRF correction from EC–EO differences.

We used ICC ≥ 0.4 as threshold. Results showed that 69.5% (49,203 voxels) and 61.7% (43,705 voxels) of the voxels in the whole brain exceeded the ICC threshold for coefficient b and power-law slope b’, respectively, under the EC condition. For the EO condition, 57.2% (40,504 voxels) and 48.4% (34,309 voxels) of the voxels exceeded the ICC threshold for the two metrics. ICC values for ALFF under EC (87.8%, 62,180 voxels for 0.01–0.1 Hz; 78.3%, 55,469 voxels for 0.01–0.25 Hz) and EO conditions (84.6%, 59,922 voxels for 0.01–0.1 Hz; 88.3%, 62,521 voxels for 0.01–0.25 Hz) were comparably higher. ICC histograms are shown in Figures 12A,B. ICC distributions of the PSS in the brain are shown in Supplementary Figures 7A,B.

In addition, we calculated the number of voxels with ICC ≥ 0.4 within the conjunction mask of significant EC–EO difference using coefficient b, power-law slope b’, and ALFF (0.01–0.1 and 0.01–0.25 Hz) combined. In total, there were 7,953 voxels in the conjunction mask. Results showed that 76.3 (6,070 voxels) and 70.5% (5,604 voxels) of the voxels exceeded the ICC threshold for coefficient b and power-law slope b’, respectively, under the EC condition. For the EO condition, 54.7 (4,354 voxels) and 44.4% (3,530 voxels) of the voxels exceeded the ICC threshold for the two measurements. ICC values for ALFF under EC (91.1%, 7,244 voxels for 0.01–0.1 Hz; 79.9%, 6,361 voxels for 0.01–0.25 Hz) and EO conditions (81.2%, 5,460 voxels for 0.01–0.1 Hz; 87.1%, 6,927 voxels for 0.01–0.25 Hz) were comparably higher. ICC histograms are shown in Figures 12C,D. ICC distributions in the brain are shown in Supplementary Figures 7C,D.

The summary of test–retest reliability results for group Z-transformation is shown in Table 2.

**TABLE 2:** Test–retest reliability with group Z-transformation.

Method	Whole brain: 70,831 voxels				Conjunction mask: 7,953 voxels			
	EC		EO		EC		EO	
PSS b	49,203	69.5%	40,504	57.2%	6,070	76.3%	4,354	54.7%
PSS b’	43,705	61.7%	34,309	48.4%	5,604	70.5%	3,530	44.4%
ALFF-0.1 Hz	62,180	87.8%	59,922	84.6%	7,244	91.1%	5,460	81.2%
ALFF-0.25 Hz	55,469	78.3%	62,521	88.3%	6,361	79.9%	6,927	87.1%

### Intra-Class Correlation for Eyes Closed and Eyes Open Using Individual Z-Transformation

**FIGURE 13:**
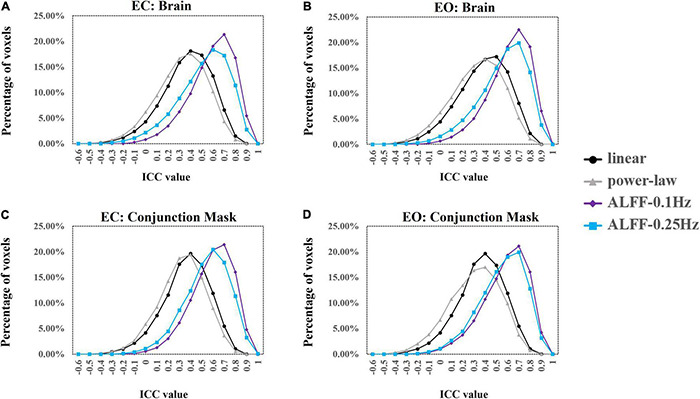
ICC histograms of coefficient b and power-law slope b’ and ALFF under EC and EO conditions using individual Z-transformation. Panels **(A,B)** show the histograms of ICC values of EC **(A)** and EO **(B)** conditions from the entire brain. Panels **(C,D)** show the histograms of ICC values of EC **(C)** and EO **(D)** conditions from the conjunction mask of the voxels that survived GRF correction from EC–EO differences.

Results showed that 47.9 (33,963 voxels) and 40.0% (28,085 voxels) of the voxels exceeded the ICC threshold for coefficient b and power-law slope b’, respectively, under the EC condition. For the EO condition, 50.3 (35,659 voxels) and 41.1% (29,136 voxels) of the voxels exceeded the ICC threshold for the two measurements. ICC values for ALFF under EC (83.0%, 58,814 voxels for 0.01–0.1 Hz; 77.6%, 55,950 voxels for 0.01–0.25 Hz) and EO conditions (86.0%, 60,885 voxels for 0.01–0.1 Hz; 72.1%, 51,093 voxels for 0.01–0.25 Hz) were comparably higher. ICC histograms are shown in Figures 13A,B. ICC distributions in the brain are shown in Supplementary Figures 8A,B.

In addition, we calculated the number of voxels with ICC ≥ 0.4 within the conjunction mask of significant EC–EO condition using coefficient b, power-law slope b’, and ALFF (0.01–0.1 Hz and 0.01–0.25 Hz) combined. In total, there were 14,103 voxels in the conjunction mask. Results showed that 45.5 (6,421 voxels) and 37.4% (5,280 voxels) of the voxels exceeded the ICC threshold for coefficient b and power-law slope b’, respectively, under the EC condition. For the EO condition, 46.2 (6,513 voxels) and 37.3% (5,258 voxels) of the voxels exceeded the ICC threshold for the two measurements. ICC values for ALFF under EC (84.2%, 11,873 voxels for 0.01–0.1 Hz; 77.7%, 10,953 voxels for 0.01–0.25 Hz) and EO conditions (81.5%, 11,490 voxels for 0.01–0.1 Hz; 77.5%, 10,923 voxels for 0.01–0.25 Hz) were comparably higher. ICC histograms are shown in Figures 13C,D. ICC distributions in the brain are shown in Supplementary Figures 8C,D.

The summary of test–retest reliability results for individual-Z-transformation is shown in Table 3.

**TABLE 3:** Test–retest reliability with individual-Z-transformation.

Method	Whole brain: 70,831 voxels				Conjunction mask: 14,103 voxels			
	EC		EO		EC		EO	
PSS b	33,963	47.9%	35,659	50.3%	6,421	45.5%	6,513	46.2%
PSS b’	28,085	40.0%	29,136	41.1%	5,280	37.4%	5,258	37.3%
ALFF-0.1 Hz	58,814	83.0%	60,885	86.0%	11,873	84.2%	11,490	81.5%
ALFF-0.25 Hz	55,950	77.6%	51,093	72.1%	10,953	77.7%	10,923	77.5%

### Intra-Class Correlation of the Parahippocampus Regions of Interest

**FIGURE 14:**
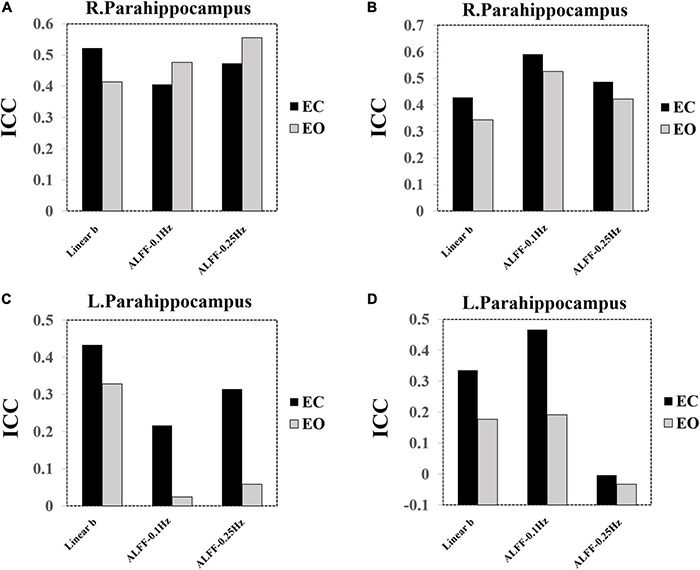
ICC bar plots of the bilateral parahippocampus. **(A)** ICC value of the right parahippocampus using group Z-transformation. **(B)** ICC value of the right parahippocampus using individual Z-transformation. **(C)** ICC value of the left parahippocampus using group Z-transformation. **(D)** ICC value of the left parahippocampus using individual Z-transformation.

As the paired *t*-tests have shown, the PSS (linear coefficient b) approach could provide exclutional difference of EC and EO conditions in the bilateral parahippocampus than the ALFF. In addition, we presented the ICC bar plots for the left and right parahippocampus ROI (left: [−27, −18, −24]; right: [24, −15, −21], radius = 5 mm) (Figure 14). PSS of linear coefficient b had higher ICC values compared with ALFF using group Z-transformation (Figures 14A,C) but lower ICC values compared with ALFF using individual Z-transformation (Figures 14B,D).

### Goodness-of-Fit of Eyes Closed and Eyes Open

The GoF was calculated based on the residuals of regression, with a larger GoF indicating better fitting. For the EC condition, the mean GoF across subjects of linear coefficient b was larger than the mean GoF of power-law slope b’ in 99.12% of the voxels. For the EO condition, the mean GoF of linear coefficient b was larger than the mean GoF of power-law slope b’ in 99.51% of the voxels. For both the EC and EO conditions, the Gof of power-law slope b’ was higher only in the orbital frontal area. The mean GoF maps of the 145 subjects between EC and EO are shown in the Supplementary Materials (Supplementary Figures 9, 10).

## Discussion

We investigated the validity and the reliability of the PSS using 145 subjects with EC and EO conditions. Two metrics, namely, linear coefficient b and power-law slope b’ for the PSS, were provided. For comparison, we also provided results for ALFF. In general, ALFF showed higher validity and reliability, but PSS could identify exclusive differences in brain areas such as the parahippocampus than ALFF. Detailed results are discussed in the following paragraphs.

### Distribution of Power Spectrum Slope in Eyes Closed and Eyes Open Conditions

We showed the individual Z-transformed PSS image with linear coefficient *b* as an example for the illustration of the spatial distribution of PSS. There was a clear boundary between the gray matter and the white matter where Z-PSS was negative (larger absolute slope than the global mean) in the gray matter and positive in the white matter and the brain stem. This result demonstrated that the power magnitude of signals decreased more in the gray matter voxels than that in the white matter and the brain stem. It could also be obtained that the visual area showed the most power decay.

### Power Decay Is Different Between Eyes Closed and Eyes Open Conditions

The amplitude of RS-fMRI signal power in the frequency domain decreases with increasing frequency bin, yet the magnitude of decrease was different between the EC and EO conditions. As shown in this study, the signal power of the sensorimotor area, the thalamus, and the temporal parietal junctions decayed more in EC than that in EO conditions while the power in the default mode network and visual area decayed less in the EC condition. These differences can be detected using both the linear coefficient b and power-law slope b’, yet the linear coefficient b provided more differences in the limbic system, such as the amygdala, the orbital frontal cortex, and the parahippocampus. Therefore, we considered that linear coefficient b as a better choice over the power-law slope b’ to distinguish EC and EO conditions. In addition, we observed that the individual-level Z-transformation provided highly similar results as the group-level Z-transformation, indicating that individual-level normalization may be sufficient for different scan protocols.

The difference of PSS in EC and EO conditions may represent different mechanisms of cortical physiological state. As illustrated in the *post-hoc* plots of the PSS results, “cross-over” effects of the power spectrum can be seen in the low-frequency range, resulting in a “deeper” curve of PSS decay in the sensorimotor area and a “shallower” curve of power decay in the occipital lobe. These results demonstrated that the power distributions were different between EC and EO conditions, indicating a state-dependent power spectrum (He, 2011). Comparably, the sensorimotor area and the thalamus have “steeper” power decay in the EC condition, suggesting that these two areas are more involved in long-range memory (Eke et al., 2002) whereas the default mode area and the visual area showed “shallower” power decay in the EC condition, indicating a more active and redundant neural processing of information (He, 2011).

### Specific Eyes Closed–Eyes Open Difference by Power Spectrum Slope as Compared to That by Amplitude of Low-Frequency Fluctuation

There was a high spatial overlap of EC and EO differences between the PSS and ALFF in the visual area, the default mode network, the thalamus, and the sensorimotor area. Whereas ALFF yielded more areas of EC–EO differences than PSS, the PSS yielded unique EC–EO differences in the brain, such as the parahippocampus. In addition, we extracted the power spectrum of the parahippocampus and showed that the “cross-over” of power magnitude is located in the high-frequency zone (>0.1 Hz). Even with a *p* < 0.01 uncorrected threshold, no voxels showed significant EC–EO difference in the parahippocampus using ALFF, indicating that PSS can capture unique EC–EO differences.

From the methodological point of view, ALFF measures the average power oscillation within the given frequency range (normally in the low-frequency band), which limits the ability to investigate how the fMRI signal power distributes differently in different frequency bands. As shown in previous studies, brain signals oscillate differently in different frequency bands of neurological and psychiatric diseases such as Parkinson’s disease (Hou et al., 2014) and schizophrenia (Yu et al., 2014). Investigating the change of BOLD oscillations in the high-frequency band in addition to the conventional 0.01–0.1 low-frequency band becomes more critical (Malinen et al., 2010; Otti et al., 2013). While most researchers measured frequency-specific brain oscillation with ALFF according to the N3L theory as Zuo et al. (2010) did, different methods of decomposing the RS-fMRI signals in the frequency domain may influence the group-level result. Therefore, in this study, using EC and EO conditions as an example, we showed the capability of the PSS approach as an integrated way of capturing the signal oscillation differences along the frequency band and provided a GUI toolkit for the calculation.

The correlation analyses between PSS and ALFF have shown that most of the voxels were anti-correlated between the two approaches, especially in the EO condition. This observation may closely link with the differences in theory between the two approaches. PSS seeks the slope of the power spectrum whereas ALFF measures the amplitude of the power spectrum in the low-frequency band. Signals with more “power” in the low-frequency band as measured by higher ALFF may have the “steeper” slope of the power spectrum with increasing frequency bins, which caused the anti-correlation. We also obtained different PSS-ALFF correlations in the brain between EC and EO conditions. Specifically, we obtained a positive correlation between PSS and ALFF in the medial temporal gyrus, prefrontal gyrus, and the small area within the parietal cortex in the EC condition, but not the EO condition. These regions were partially overlapped with the brain areas that showed significant PSS difference with EC > EO contrast. A possible explanation is that the slope of power spectrum was “steeper” in these areas in the EO condition compared to the EC condition. It further indicated that the magnitude of power was distributed more in the low-frequency band in the EO condition, resulting in a negative correlation between the PSS and the ALFF in these areas.

### Test–Retest Reliability of Power Spectrum Slope

The high-reliability property of ALFF has been demonstrated in previous studies (Zuo et al., 2010; Zhao et al., 2018). In this study, although the PSS linear coefficient b was less reliable than ALFF, there were over 60 and 70% of voxels with ICC values greater than 0.4 in the entire brain mask and the conjunction of EC–EO difference mask, respectively. The percentage of reliable voxels was lower in the EO condition, suggesting that the PSS approach favors the EC condition in terms of reliability.

Specifically, PSS linear coefficient b showed comparable reliability in the parahippocampus than that with ALFF, indicating that the PSS approach not only can provide exclusive differences between EC and EO conditions but was also comparably reliable.

### Goodness-of-Fit

We calculated the GoF using the regression residuals for both the linear coefficient b and power-law slope b’. The GoF maps for both the two PSS metrics showed clear differences between the gray matter and the white matter as well as the CSF. In addition, higher GoF can be obtained within the visual area. This result indicated that for voxels distributing in the gray matter, especially in the visual area, the power spectrum of the signals was most likely decreasing with increasing frequency bins. However, for signals from the white matter and CSF, the power spectrum may not decrease ideally with increasing frequency bins.

The average GoF across 145 subjects favored slightly the linear coefficient b than the power-law slope b’ in most of the voxels, indicating that the linear coefficient b may be a better choice over the power-law slope b’ in terms of capturing the PSS for 0.01–0.25 Hz frequency bin. However, we did not investigate the slope of higher frequency (e.g., TR = 400 ms). Future studies are needed.

### Limitations and Outlooks

There are several limitations to the current study. First, we used only the conventional TR = 2,000 ms datasets, which constrained us to further investigate the power decays at a higher frequency. Although the public EC and EO datasets provided short TR (400 ms) RS-fMRI data, the power spectrum coefficient is more complicated than that for the long TR dataset. Investigation of PSS in the short TR datasets is needed in the future. Second, the application of the PSS approach was performed on the resting-state fMRI data. Future study is encouraged to test the capability of task fMRI data.

## Conclusion

We tested the validity and reliability of the PSS approach with both the linear coefficient b and power-law slope b’. The linear coefficient b showed higher validity and reliability than the power-law slope b’. Although the ALFF approach had higher validity and reliability than the PSS approach, the PSS approach can identify brain regions such as the orbital frontal cortex and the parahippocampus that did not show significant differences between EC and EO conditions using ALFF. These results demonstrated that the PSS approach may compensate ALFF as an alternative power-spectrum analytic approach to measure brain local activity.

## Data Availability Statement

The original contributions presented in the study are included in the article/Supplementary Material, further inquiries can be directed to the corresponding author. The PSS toolkit is available at: http://www.restfmri.net/forum/node/2760.

## Ethics Statement

This study used public datasets. The datasets were approved by the ethic committees of Beijing Normal University and Hangzhou Normal University. The patients/participants provided their written informed consent to participate in this study.

## Author Contributions

ZZ: study design, data analyses, and manuscript writing. YQ: GUI implementation. SY: manuscript revision and critical review. JL: study design and critical review. All authors contributed to the article and approved the submitted version.

## Conflict of Interest

The authors declare that the research was conducted in the absence of any commercial or financial relationships that could be construed as a potential conflict of interest.

## Publisher’s Note

All claims expressed in this article are solely those of the authors and do not necessarily represent those of their affiliated organizations, or those of the publisher, the editors and the reviewers. Any product that may be evaluated in this article, or claim that may be made by its manufacturer, is not guaranteed or endorsed by the publisher.

## Footnotes

1
https://www.nitrc.org/projects/eceo_rsfmri_9/
