# Supplementary material for: Reliability and Validity of Power Spectrum Slope (PSS): A Metric for Measuring Resting-State Functional Magnetic Resonance Imaging Activity of Single Voxels
Source: Front Neurosci. 2022 May 6;16:871609. doi: 10.3389/fnins.2022.871609 (PMC9121130; doi:10.3389/fnins.2022.871609)

Reliability and validity of power spectrum slope (PSS): a metric for the measurement of resting-state fMRI of single voxels

Supplementary Information

Table S1: Data acquisition parameters.

Dataset	RS-fMRI	3D T1
Dataset 1-3	TR/TE = 2000/30 ms, FA= 90°, 43 slices with interleaved acquisition; slice thickness/gap = 3.2/0 mm; matrix = 64 × 64; FOV = 220 × 220 mm^2; in-plane voxel size = 3.44 × 3.44 mm^2; total duration = 8 min.	176 sagittal slices, thickness = 1 mm, TR/TE = 8100/3.1 ms, FA = 9°, FOV = 250 × 250 mm^2
Dataset 4	TR/TE = 2000/30 ms, FA = 60°, 37 slices with interleaved acquisition; thickness/gap = 3.4/0 mm, matrix = 64 × 64; FOV = 220×220 mm^2; in-plane voxel size = 3.44×3.44 mm^2; total duration = 8 min	176 sagittal slices, thickness = 1 mm, TR/TE = 8100/3.1 ms, FA = 8°, FOV = 250×250 mm^2
Dataset 6	TR/TE = 2000/30 ms, FA = 90°, 33 slices with interleaved acquisition; thickness/gap = 3.5/0 mm, matrix = 64 × 64; FOV = 200×200 mm^2; in-plane voxel size = 3.1×3.1 mm^2; total duration = 8 min	128 sagittal slices, thickness = 1.33 mm, TR/TE = 2530/3.39 ms, FA = 7°, FOV = 256×256 mm^2
Dataset 7	TR/TE = 2000/30 ms, FA= 90°, 43 slices with interleaved acquisition; slice thickness/gap = 3.2/0 mm; matrix = 64 × 64; FOV= 220 × 220 mm^2; in-plane voxel size = 3.44 × 3.44 mm^2; total duration = 8 min.	176 sagittal slices, thickness = 1 mm, TR/TE = 8.1/3.1 ms, FA = 9°, FOV = 250 × 250 mm^2
Dataset 8	TR/TE = 2000/30 ms, FA = 90°, 33 slices with interleaved acquisition; thickness/gap = 3.6/0 mm, matrix = 64 × 64; FOV = 200×200 mm^2; in-plane voxel size = 3.125×3.125 mm^2; total duration = 8 min	128 sagittal slices, thickness = 1.33 mm, TR/TE = 2530/3.39 ms, FA = 7°, FOV = 256×256 mm^2

Figure S1. Spatial overlap of EC – EO differences between PSS and ALFF (0.01 - 0.1 Hz). **Panel A** shows the contrast between linear coefficient b and ALFF with group Z-transformation. **Panel B** shows the contrast between linear coefficient b and ALFF with individual Z-transformation. **Panel C** shows the contrast between power-law slope b' and ALFF with group Z-transformation. **Panel D** shows the contrast between power-law slope b' and ALFF with individual Z-transformation.

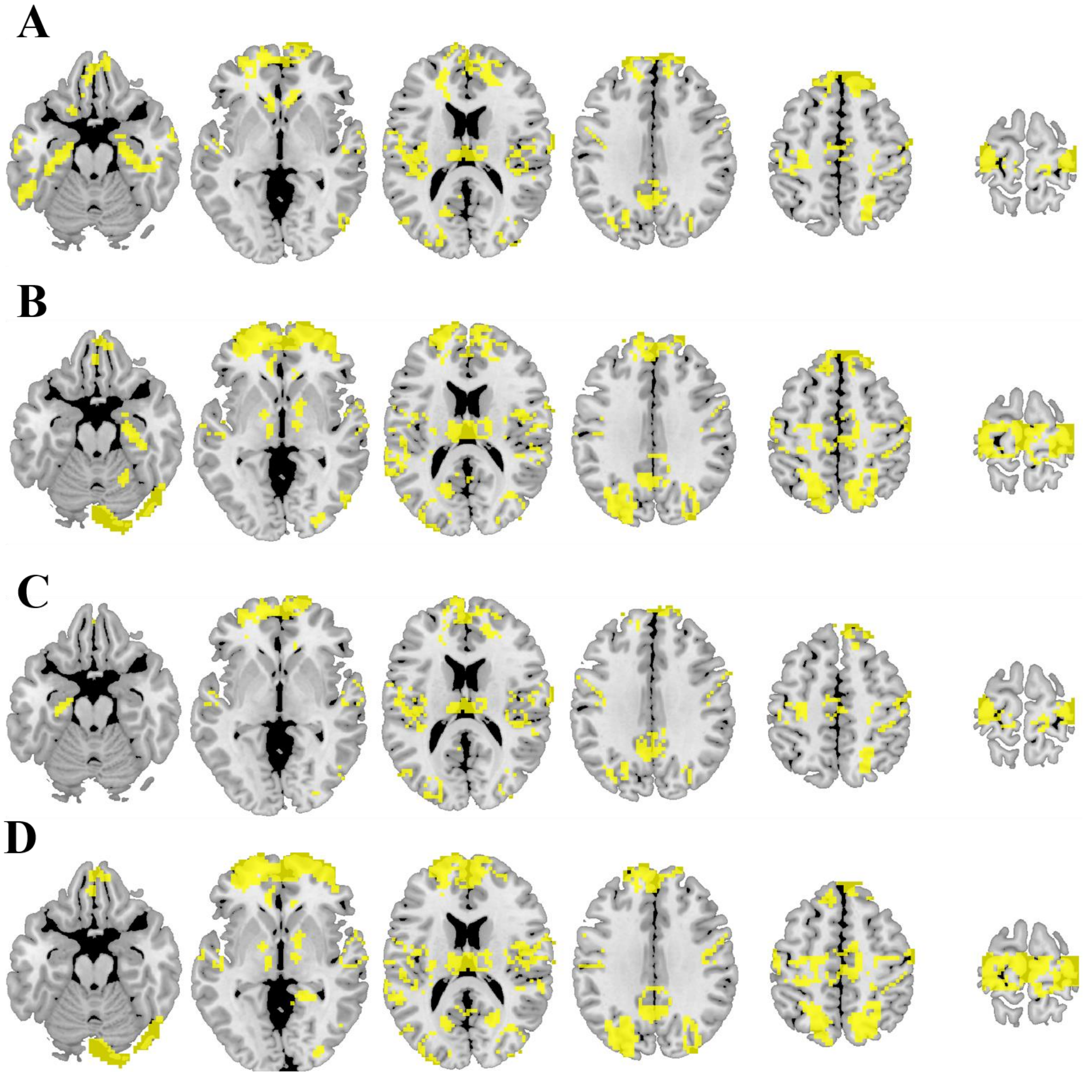

Figure S2. Spatial overlap of EC – EO differences between PSS and ALFF (0.01 - 0.25 Hz). **Panel A** shows the contrast between linear coefficient b and ALFF with group Z-transformation. **Panel B** shows the contrast between linear coefficient b and ALFF with individual Z-transformation. **Panel C** shows the contrast between power-law slope b’ and ALFF with group Z-transformation. **Panel D** shows the contrast between power-law slope b’ and ALFF with individual Z-transformation.

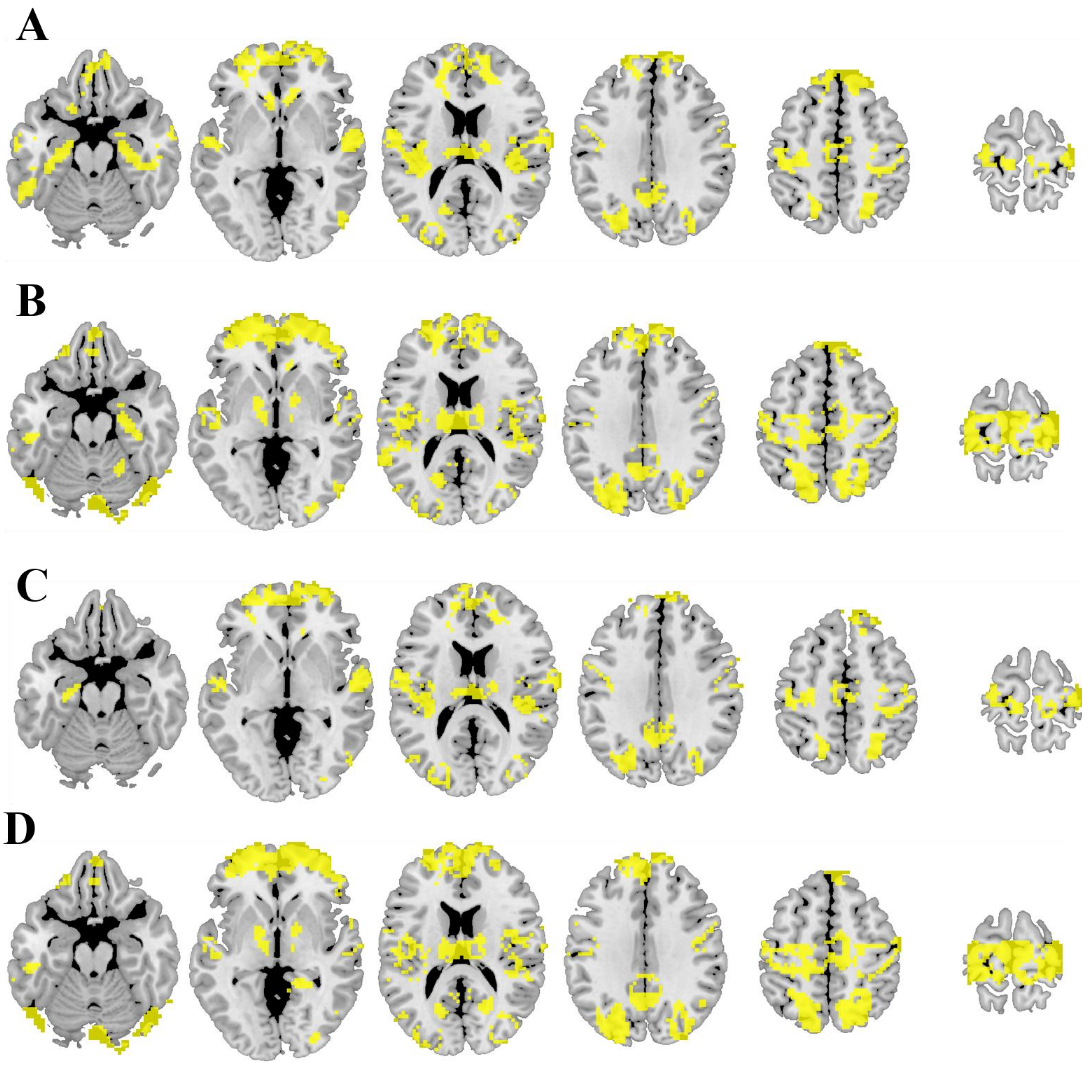

Figure S3. Intra-subject voxel-wise correlation between PSS and ALFF (0.01 – 0.1 Hz) in EC condition. **Panel** A shows the correlation of linear coefficient b and ALFF. **Panel B** shows the correlation of power-law slope b' and ALFF.

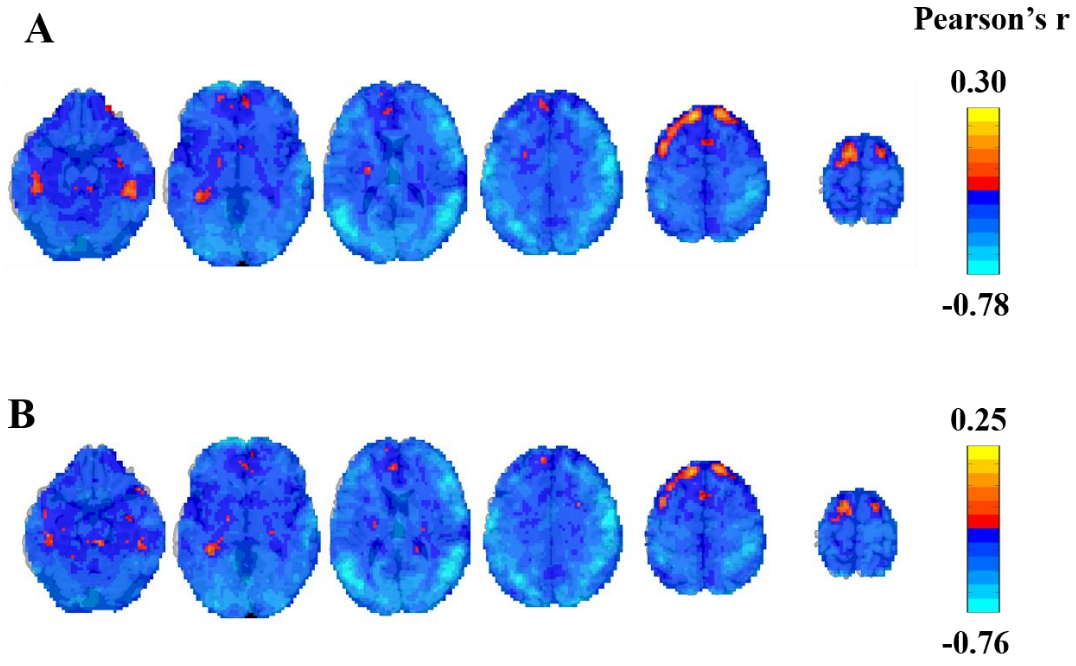

Figure S4. Intra-subject voxel-wise correlation between PSS and ALFF (0.01 – 0.1 Hz) in EO condition. **Panel** A shows the correlation of linear coefficient b and ALFF. **Panel B** shows the correlation of power-law slope b’ and ALFF.

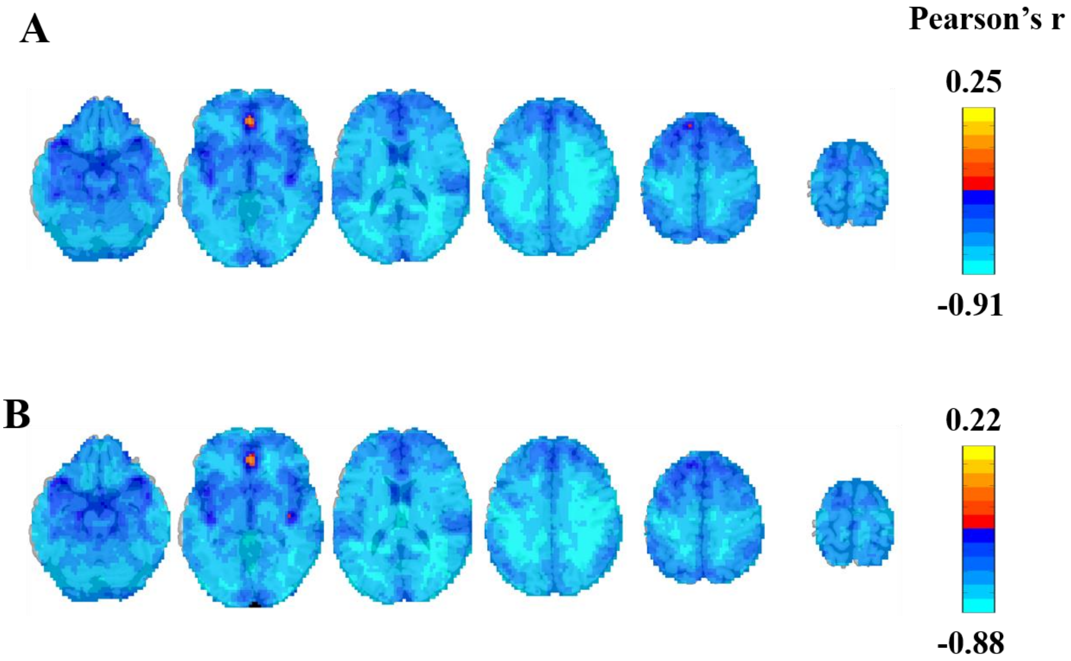

Figure S5. Intra-subject voxel-wise correlation between PSS and ALFF (0.01 – 0.25 Hz) in EC condition. **Panel** A shows the correlation of linear coefficient b and ALFF. **Panel B** shows the correlation of power-law slope b’ and ALFF.

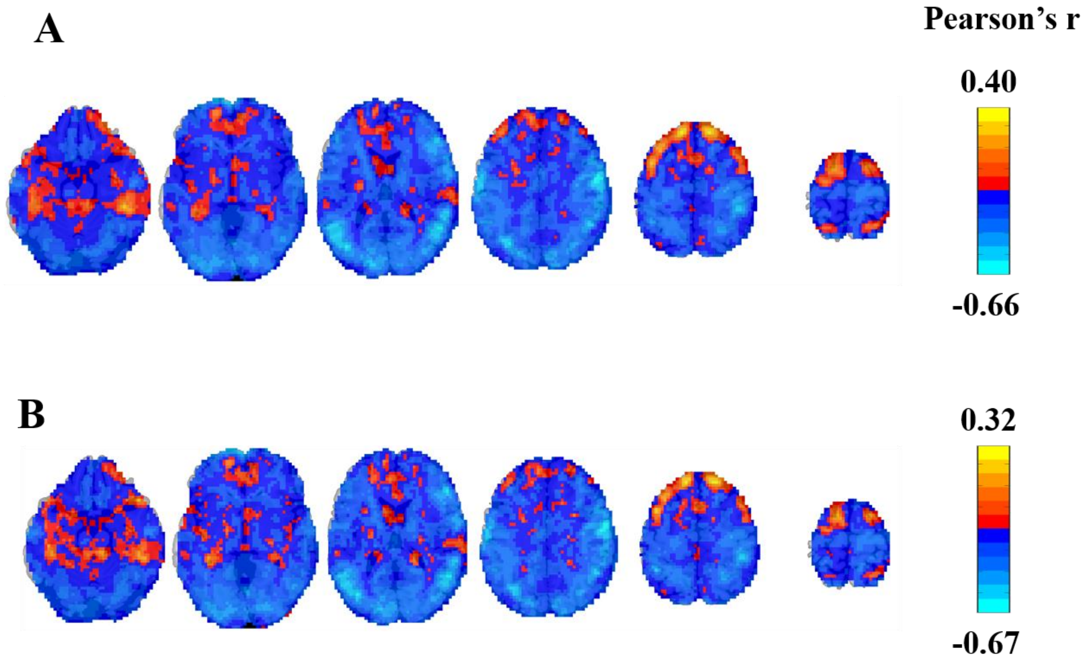

Figure S6. Intra-subject voxel-wise correlation between PSS and ALFF (0.01 – 0.25 Hz) in EO condition. **Panel** A shows the correlation of linear coefficient b and ALFF. **Panel B** shows the correlation of power-law slope b' and ALFF.

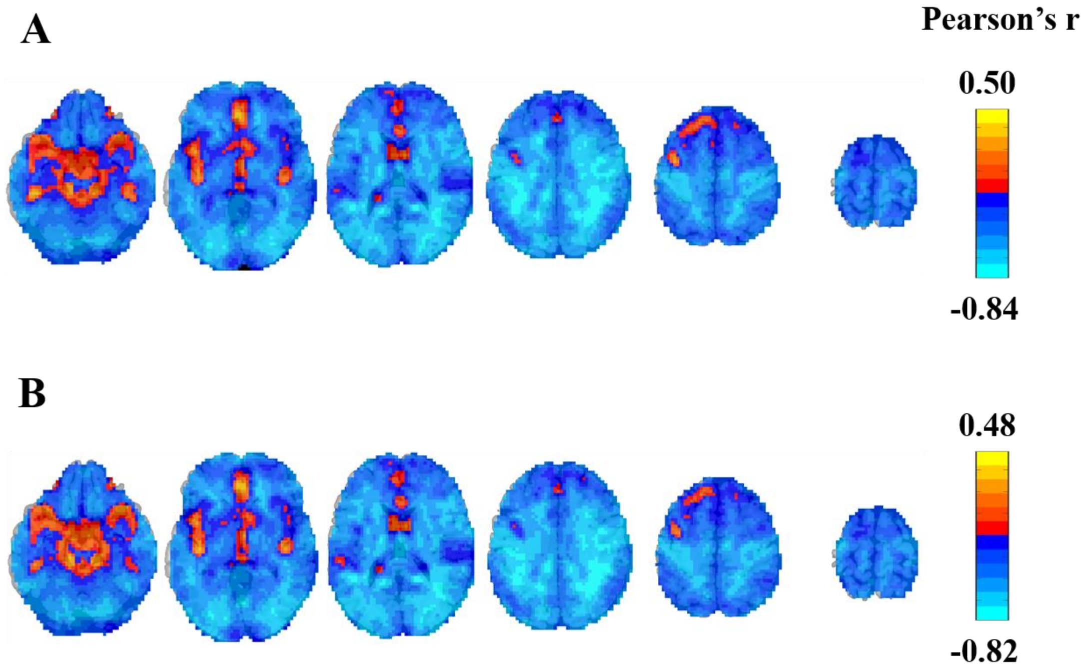

Figure S7. ICC distribution of PSS within group-Z transformation **Panel A**: linear coefficient b of EC. **Panel B**: power-law slope b' of EC. **Panel C**: linear coefficient b of EO. **Panel D**: power-law slope b' of EO.

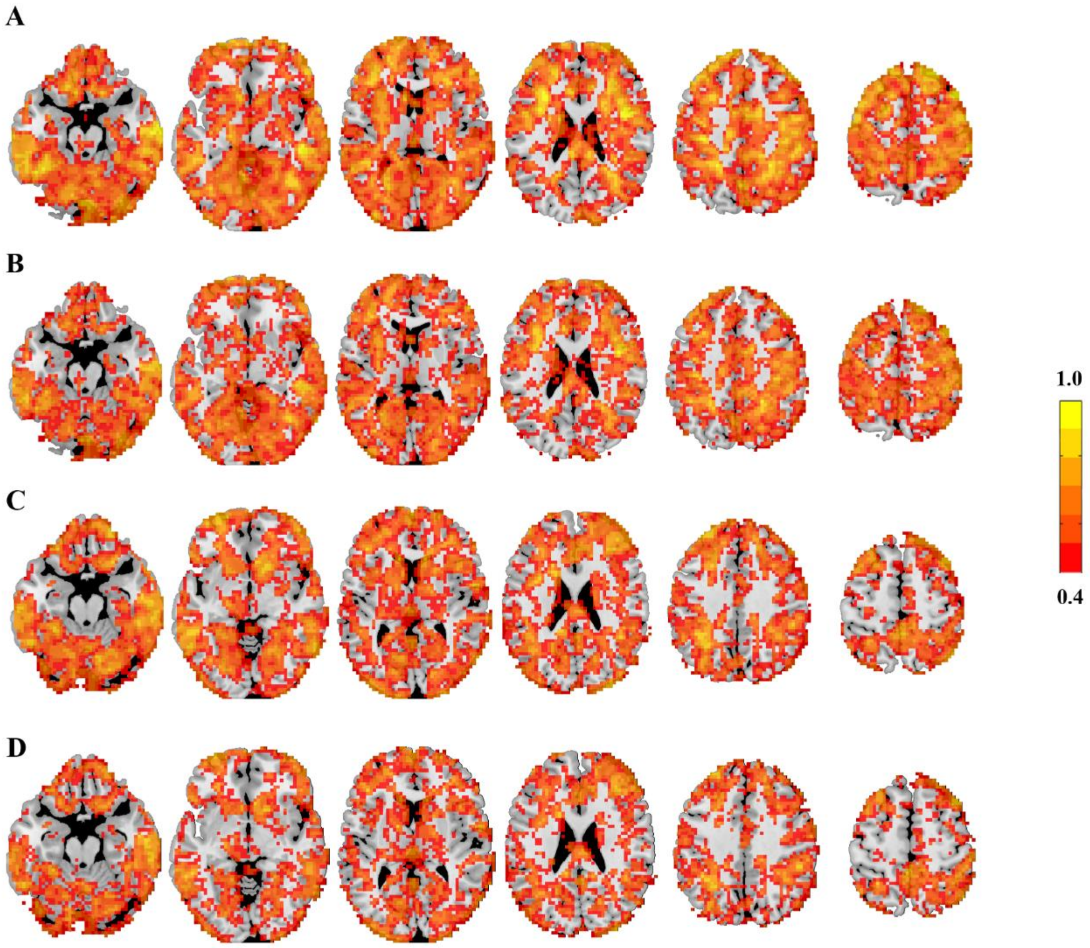

Figure S8. ICC distribution of PSS within individual-Z transformation. **Panel A**: linear coefficient b of EC. **Panel B**: power-law slope b' of EC. **Panel C**: linear coefficient b of EO. **Panel D**: power-law slope b' of EO.

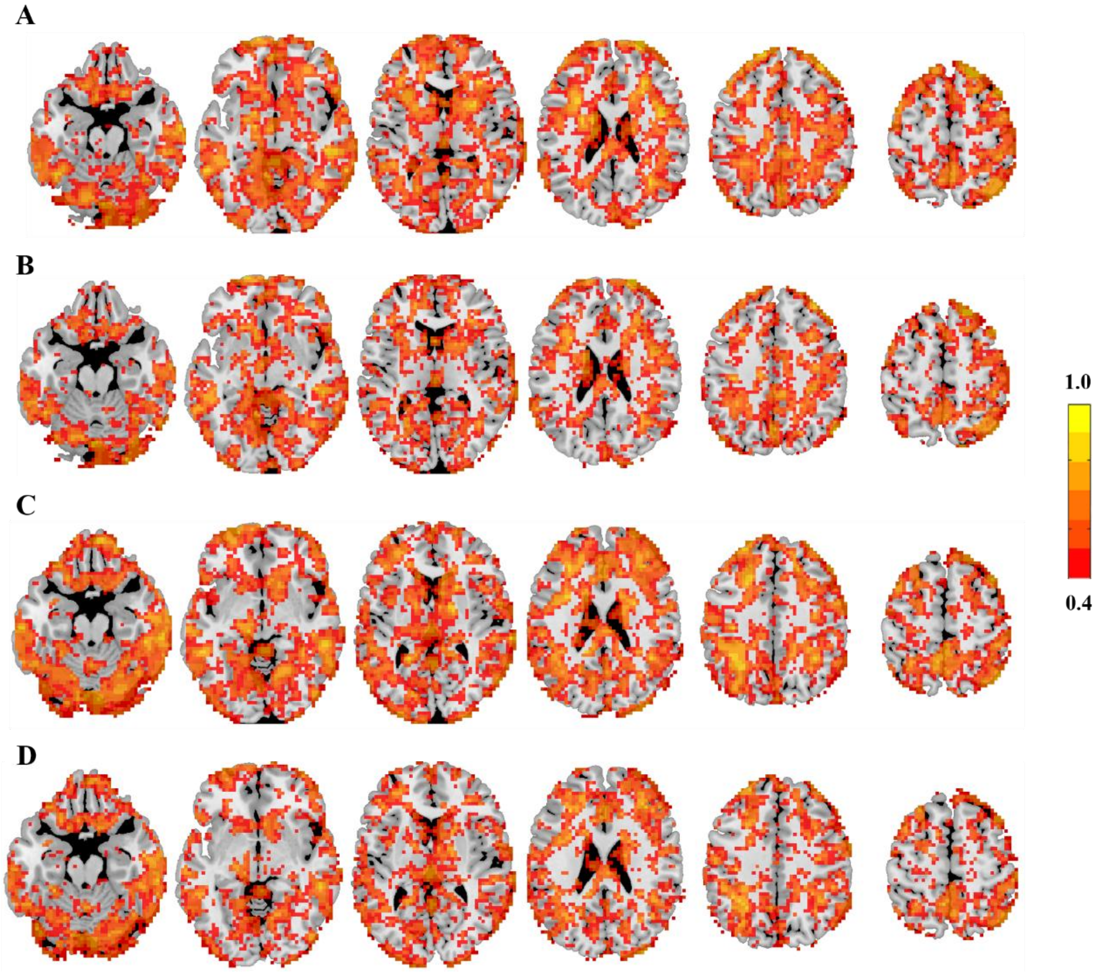

Figure S9. Goodness-of-fit of EC condition. **Panel A** is for linear coefficient b. **Panel B** is for power-law slope b'. **Panel C** is for b-b' difference.

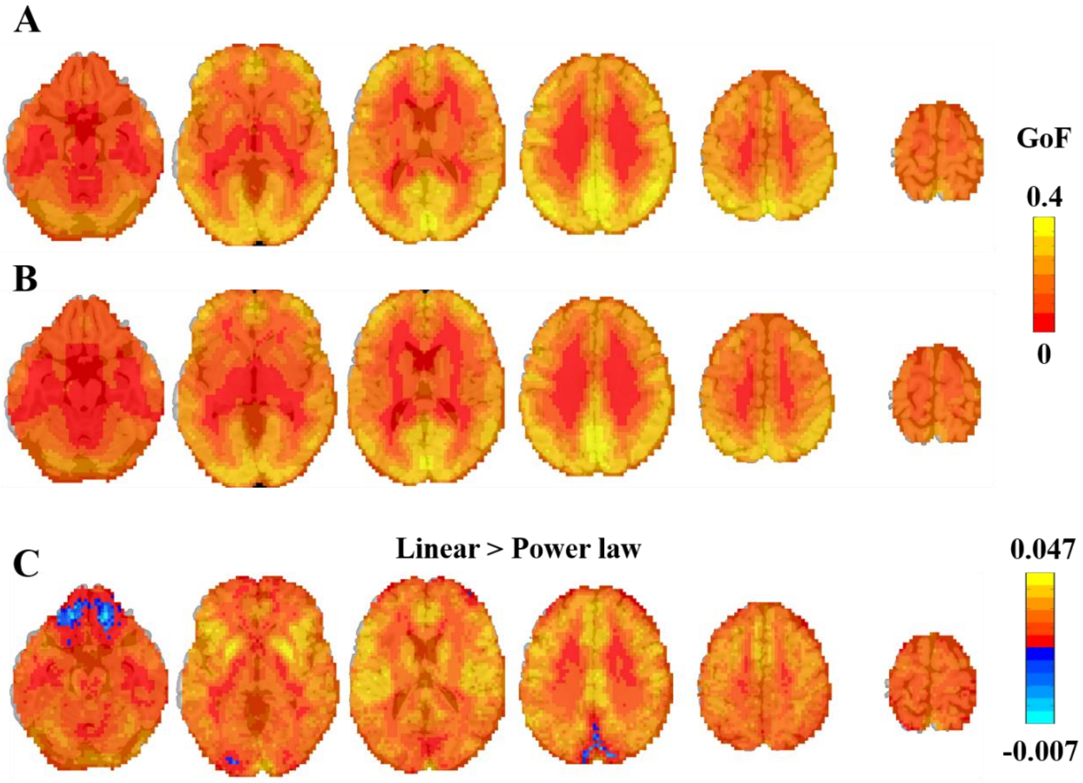

Figure S10. Goodness-of-fit of EO condition. **Panel A** is for linear coefficient b. **Panel B** is for power-law slope b'. **Panel C** is for b-b' difference.

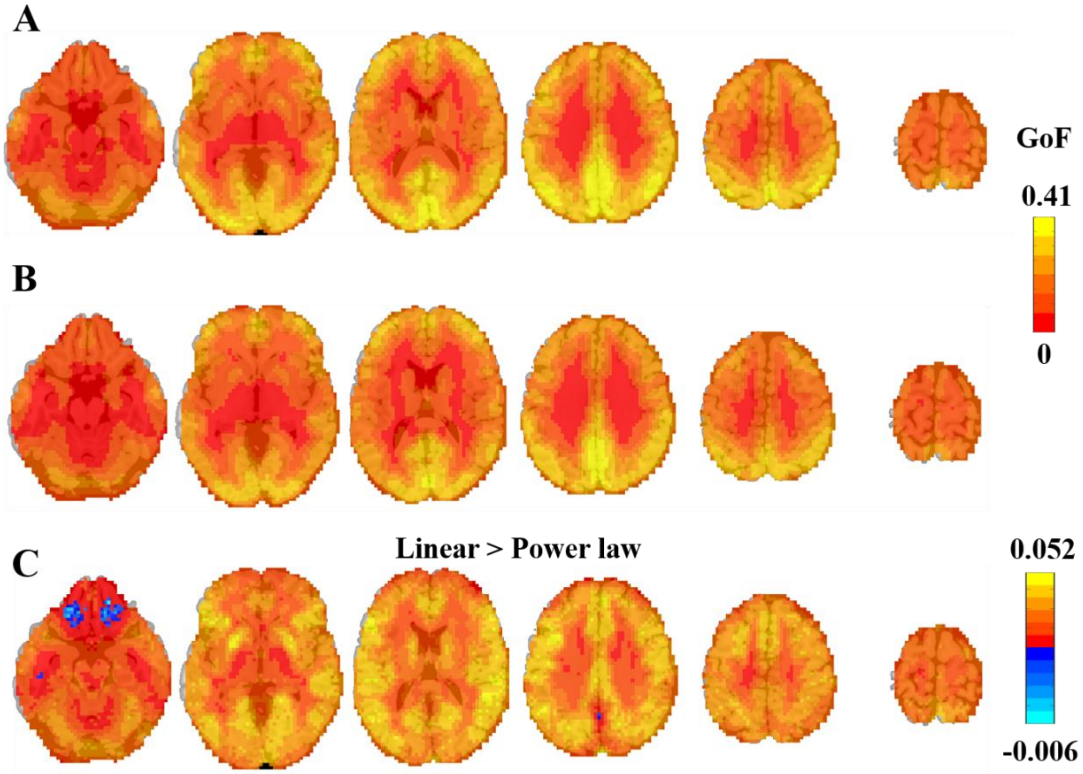

Supplement: Supplementary file 1 [file Data_Sheet_1.PDF]
